# Dynamic Alterations to α-Actinin Accompanying Sarcomere Disassembly and Reassembly during Cardiomyocyte Mitosis

**DOI:** 10.1371/journal.pone.0129176

**Published:** 2015-06-15

**Authors:** Xiaohu Fan, Bryan G. Hughes, Mohammad A. M. Ali, Woo Jung Cho, Waleska Lopez, Richard Schulz

**Affiliations:** 1 Department of Pediatrics, Faculty of Medicine & Dentistry, University of Alberta, Edmonton, Alberta, Canada; 2 Department of Pharmacology, Faculty of Medicine & Dentistry, University of Alberta, Edmonton, Alberta, Canada; 3 Cardiovascular Research Centre, Faculty of Medicine & Dentistry, University of Alberta, Edmonton, Alberta, Canada; 4 Mazankowski Alberta Heart Institute, University of Alberta, Edmonton, Alberta, Canada; 5 Department of Medical Microbiology & Immunology, Faculty of Medicine & Dentistry, University of Alberta, Edmonton, Alberta, Canada; Inner Mongolia University, CHINA

## Abstract

Although mammals are thought to lose their capacity to regenerate heart muscle shortly after birth, embryonic and neonatal cardiomyocytes in mammals are hyperplastic. During proliferation these cells need to selectively disassemble their myofibrils for successful cytokinesis. The mechanism of sarcomere disassembly is, however, not understood. To study this, we performed a series of immunofluorescence studies of multiple sarcomeric proteins in proliferating neonatal rat ventricular myocytes and correlated these observations with biochemical changes at different cell cycle stages. During myocyte mitosis, α-actinin and titin were disassembled as early as prometaphase. α-actinin (representing the sarcomeric Z-disk) disassembly precedes that of titin (M-line), suggesting that titin disassembly occurs secondary to the collapse of the Z-disk. Sarcomere disassembly was concurrent with the dissolution of the nuclear envelope. Inhibitors of several intracellular proteases could not block the disassembly of α-actinin or titin. There was a dramatic increase in both cytosolic (soluble) and sarcomeric α-actinin during mitosis, and cytosolic α-actinin exhibited decreased phosphorylation compared to sarcomeric α-actinin. Inhibition of cyclin-dependent kinase 1 (CDK1) induced the quick reassembly of the sarcomere. Sarcomere dis- and re-assembly in cardiomyocyte mitosis is CDK1-dependent and features dynamic differential post-translational modifications of sarcomeric and cytosolic α-actinin.

## Introduction

Lower vertebrate animals such as amphibians and teleost fish retain a remarkable capacity for cardiac regeneration throughout life [1,2]. Adult zebrafish can regenerate their heart without scar formation even after 20% of the ventricle is resected [3]. However, adult mammals obviously lack this full regenerative capacity. Therefore lesions in the myocardial infarction zone can only be repaired by fibrotic scarring, which leads to heart insufficiency and accounts for the high rate of morbidity and mortality resulting from ischemic heart disease.

The heart is the first functional organ that develops during the embryogenesis of vertebrates [4]. During mouse heart development, embryonic cardiomyocytes develop intracellular myofibrils and begin contracting on embryonic day 8.5 [5]. Multiple sarcomeric proteins are sequentially assembled into a complex contractile apparatus, with the sarcomere being its most basic unit, to generate the force needed for contraction [6]. Embryonic cardiomyocytes quickly proliferate and cell division is accompanied by special structural modifications which involve two main sequential steps. First, myofibrillar disassembly enables chromosome segregation and remodeling of various subcellular components to accomplish a complete cell division cycle [7]. In this step, cardiomyocytes stop contracting but retain their intercellular contacts. Next, myofibrils reassemble after cell division and contraction resumes [7]. Understanding these complex processes might provide a key as to why postnatal cardiomyocytes stop dividing and instead undergo hypertrophy in response to physiological or pathological challenges after birth [8].

Because the sarcomere occupies a large volume of the mature cardiomyocyte, it physically impedes mitosis and cytokinesis. Sarcomere disassembly is a prerequisite task for cardiomyocyte proliferation [7]. This allows one to speculate that the limited regeneration capacity of the mammalian heart beginning in early postnatal life may be attributed to the increasing maturity and complexity of sarcomere structure and the onset of the hypertrophic mechanism. Indeed, the off-switch of proliferative capacity in the mouse heart is coincident with the start of cardiomyocyte hypertrophy [9] and binucleation. Cardiomyocytes in the one day old neonatal mouse heart are predominantly mononucleated (99%) with almost no binucleated cells. Interestingly, just 8–9 days after birth, more than 98% of mouse cardiomyocytes become binucleated while losing their proliferative capacity at the same time [10]. The emergence of binucleated cardiomyocytes could be interpreted as successful karyokinesis followed by failed cytokinesis attributable to insufficient myofibril disassembly in the last cell cycle of the post-neonatal cardiomyocyte [11,12], although there is also evidence that it is instead due to a cytoskeletal defect resulting in incomplete closure of the actomyosin contractile ring [13].

The mechanism of sarcomere disassembly remains poorly understood thus far. We hypothesized that intracellular proteases may facilitate disassembly by proteolysis of key sarcomeric proteins. In the cardiovascular system, the matrix metalloproteinases (MMPs), especially MMP-2, are abundantly expressed in cardiomyocytes [14]. Besides the well-known extracellular localization and substrates of MMP-2, it is also a bona fide intracellular protease [15] which is also localized to specific subcellular compartments in the cardiomyocyte, including the sarcomere [14] and nucleus [16]. Upon its direct activation by increased oxidative stress [17,18] MMP-2 cleaves specific intracellular proteins including its substrates in the sarcomere such as α-actinin [19], troponin I [14], myosin light chain-1 [20], titin [21] and GSK-3β [22]. Indeed, MMP-2 has a preferential localization to the Z-disc region [21] of the cardiomyocyte which is a proposed locus of the disassembly process [7]. Thus, MMP-2 joins other intracellular proteases such as calpain, serine proteases and the proteasome system which may be candidate proteases in sarcomere disassembly. Alternatively, a second hypothesis is that post-translational modifications, such as altered phosphorylation status of key sarcomeric proteins, may lead to the remodeling of sarcomeric structure.

Most studies in this field have mainly focused on documenting the changes in regulation of the cell cycle and attempts to reactivate it to revive regenerative capacity of heart [23,24]. There have been a small number of studies examining sarcomere disassembly [7,13]. While these have established rough timelines of the process, showing that Z-band-associated proteins tend to disassemble earlier in mitosis relative to proteins associated with the M-band, the mechanistic aspects of the process remain largely unknown. However, it has been reported that proteosome inhibition in embryonic cardiomyocytes prevents disassembly of sarcomeric α-actinin during mitosis [7]. This supports our hypothesis that this could be, at least in part, a process driven by proteolysis.

In this study, we systemically analyzed the process of sarcomere disassembly by focusing on the dynamics of the sarcomere scaffold protein/molecular spring titin [25,26] and the Z-disk anchoring protein α-actinin [27,28]. Sarcomeric α-actinin (ACTN2) is an actin-binding protein which belongs to the spectrin superfamily [27,28]. In striated cardiac muscle cells it is localized at the Z-disks where it stabilizes the muscle contractile apparatus by forming a lattice-like structure which anchors the actin filaments [29]. Besides cross-linking actin filaments, α-actinin associates with a number of cytoskeletal and signaling molecules, the cytoplasmic domains of transmembrane receptors and ion channels, giving it important structural and regulatory roles in the organization of the cytoskeleton and muscle contraction [30]. We examined the mechanism of sarcomere disassembly by evaluating the possible role of intracellular proteases such as MMP-2, serine proteases, calpains and the proteasome system, and also by studying the possible role of non-proteolytic protein modifications during mitosis.

## Materials and Methods

### Neonatal rat ventricular myocytes isolation and culture

This study was conducted according to the Guide to the Care and Use of Experimental Animals published by the Canadian Council on Animal Care, and was approved by the Animal Care and Use Committee of the University of Alberta. Neonatal rat ventricular myocytes (NRVM) were isolated and cultured from 1- to 2-day-old Sprague–Dawley rats. Hearts from the rat pups were removed and the ventricles were minced and digested with collagenase II (0.10% w/v), trypsin (0.05% w/v) and DNase (0.025% w/v) in phosphate-buffered saline at 37°C for 20 min. After digestion the tissue was centrifuged at 200 x g for 1 min at 4°C in 20 ml of DMEM-F12 media (Sigma) containing 20% fetal bovine serum (Invitrogen), and 50 μg/ml gentamycin. The first supernatant was discarded and the pellet was subsequently added to the DNase/collagenase/trypsin buffer for further digestion at 37°C for 20 min. After a second digestion and centrifugation, the supernatant was collected and subjected to a third digestion. Collected supernatants were pooled and centrifuged at 300 x g for 7 min at 4°C. The resulting pellet was resuspended in 10 ml of culture medium (DMEM-F12 containing 10% fetal bovine serum and 50 μg/ml gentamycin) and the cell suspension was filtered through a cell strainer (BD Biosciences) and pre-plated for 60–90 min at 37°C to allow fibroblasts to attach. Non-adherent cells were removed and added to 35 mm dishes (Falcon) at a density of 1.8–2.0 x 10^6^ cells/dish and incubated at 37°C in culture media.

### Lentiviral vector production and transduction of cardiomyocytes

Mouse α-actinin-2 cDNA lacking a stop codon was subcloned into a lentiviral construct containing a C-terminal Halotag (Promega) fragment fused to the insert. Lentiviral vectors were produced by transient transfection of 293T cells with the 3^rd^ generation (4 plasmid) system. For transduction of cardiomyocytes, 1X10^6^ neonatal cardiomyocytes were incubated for 8 hr with 5X10^6^ transduction units of lentiviral vector in the presence of 6 μg/ml polybrene (Invitrogen) in culture medium. The medium was then replaced with fresh medium, and the cells were incubated for 72 hr prior to assays.

### Immunofluorescence and live cell imaging

Cells were washed with PBS and fixed with 4% paraformaldehyde for 20 min. After washing with PBS, cells were permeabilized for 1 min with 0.25% Triton X-100, 0.2% BSA in PBS. Cells were then incubated with primary antibodies (1:1000 dilution), including anti-α-actinin (Abcam ab9465) and anti-titin (M8 and T12; [31,32]). Then, secondary antibodies conjugated to either AlexaFluor 488 or 568 (Invitrogen) (1:2000 dilution) in PBS, 0.2% BSA for 1 hr each, and then washed three times with PBS. After the final wash, cells were mounted in ProLong Antifade with DAPI (Invitrogen). The images were acquired using a 60X oil objective (Olympus 60X/1.42) on a multi-channel spinning disc confocal microscope (1X81F-3; Olympus) and analyzed with Volocity 6.1.1 (PerkinElmer).

**Fig 1:**
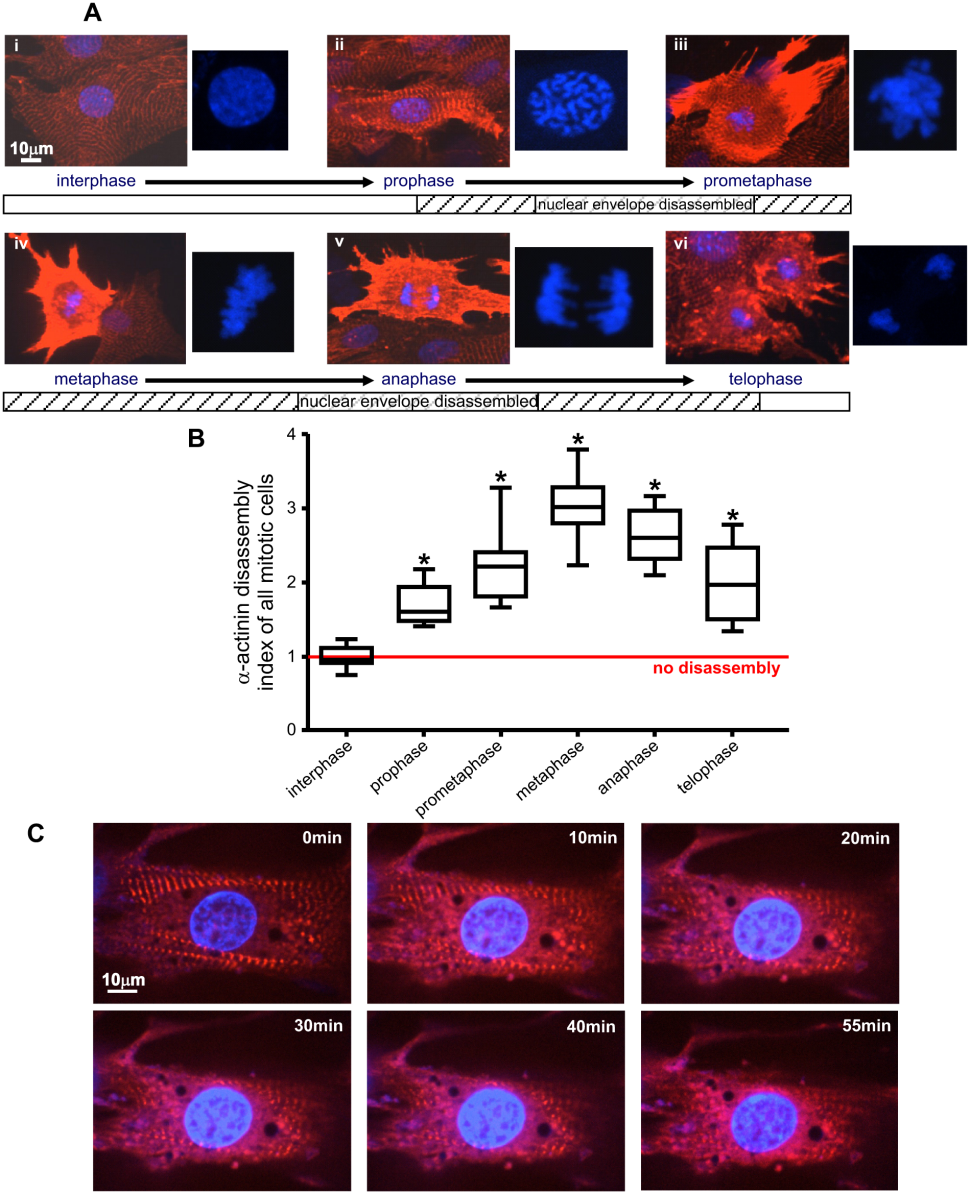
Changes in native sarcomeric α-actinin during mitosis of NRVM. (A) Immunofluorescence staining of sarcomeric α-actinin (red) in NRVM during different mitotic stages (interphase to telophase, i–vi, as indicated below each panel). DNA is stained with DAPI (blue), and this is shown alone and in close-up to the right of each panel. An ordered sarcomeric striation pattern of α-actinin staining is observed in interphase. Sarcomere disassembly begins in prophase as seen by a loss in striations and an increase in a more diffuse and higher intensity fluorescence throughout the cytoplasm. The disassembly of α-actinin peaks in metaphase, continues in anaphase, and is still observable during telophase. Reassembly starts in late telophase/cytokinesis in which the striated pattern of sarcomeric α-actinin starts to re-appear from the margin of cells. (B) Changes in the α-actinin disassembly index during NRVM mitosis. Immunofluorescence staining of α-actinin in NRVM at different mitotic stages were recorded with Volocity 6.1.1 via confocal microscopy. For each sub-phase of mitosis, 20 mitotic cells randomly chosen from our collected image library (from three independent cell isolations) were compared with 20 neighboring, interphase cells to determine the α-actinin disassembly index. The α-actinin disassembly index was calculated as mean AlexaFluor 568 intensity of the mitotic cell divided by that of an interphase cell inside the same field of view and presented as the median in a box and whisker plot. One way ANOVA followed by Tukey's multiple comparison test was performed to analyze differences between each mitotic phase. * p < 0.01 compared to interphase. (C) Time lapse live-cell images show the rapid progress of α-actinin disassembly during early prophase. NRVM were transduced with a lentiviral vector harboring C-terminal HaloTag fused α-actinin. The cells were stained with cell permeable TMRDirect halo ligand (red) 48 hr after transduction. Nuclear DNA was stained with Vybrant DyeCycle Violet (blue). The cell was observed in a live cell imaging chamber (37°C) supplied with 5% CO_2_ in room air and imaged using spinning disk confocal microscopy. Photos selected at the indicated times are displayed. Original video available in the supplementary video.

The stage of mitosis was determined by DAPI staining, and only cells showing a typical chromosomal arrangement for each phase, as described below, were used (Fig 1A). In prophase, the nuclear chromatin starts to become organized and condenses into thick strands that eventually become observable as chromosomes. However, the round nuclear envelope still remains intact. In prometaphase, chromosomes appear spread out and the nucleus region transforms from a round to an irregular shape, corresponding to loss of the nuclear envelope. In metaphase the chromosomes are aligned in a single plane midway between the spindle poles. In anaphase, the two chromatids from each chromosome are pulled apart and have migrated to the opposite spindle poles. In telophase, the daughter chromosomes have arrived at the spindle poles. Individual chromosomes start to become less clearly defined as they begin to de-condense back into chromatin.

For live cell imaging, NRVM grown on glass cover slips (25 mm in diameter) were transduced for 8 hr with lentiviral vector harboring C-teminal HaloTag labeled α-actinin. Forty-eight hr after lentiviral transduction NRVM were exposed to cell permeable TMRDirect ligand (Promega) and Vybrant DyeCycle Violet for 2 hr. Chambered cover slips were set up in a live cell, top-stage incubator chamber (Live Cell Instruments) supplied with 5% CO_2_ at 37°C, and time lapse images were taken every 60 s on a spinning disc confocal microscope (1X81F-3; Olympus). Repeated laser illumination of cells over an extended period of time resulted in cell death, and consequently we were not able to monitor any given cell for more than short phases of the binucleation/division process.

### Determination of α-actinin disassembly index

Immunofluorescent staining of native sarcomeric α-actinin in cultured NRVM was performed as described above, and images of NRVM at different mitotic stages were recorded with Volocity 6.1.1. 20 mitotic cells in each sub-phase of mitosis were compared with 20 neighboring, interphase cells. Analyzed cells were randomly chosen from our collected image library (from three independent cell isolations). Each selected cell was manually outlined using the freehand cursor and mean AlexaFluor 568 intensity relative to surface area was measured by Volocity 6.1.1. The α-actinin disassembly index was calculated as mean AlexaFluor 568 intensity of the mitotic cell divided by that of an interphase cell within the same field.

### Simultaneous assessment of α-actinin and titin disassembly scores for protease inhibitor treatments

NRVM were cultured in the presence of different protease inhibitors (PMSF, 20 μM; ARP100, 100 μM; GM6001, 5 μM; ALLM, 100 μM; MG132, 10 μM) for 4 hr before they were processed for immunofluorescence staining of α-actinin, the M8 epitope of titin and DAPI. All inhibitors were used at a pre-determined highest concentration that did not cause obvious visual cell damage during 4 hr exposure. Screening for mitotic cells based on DNA-DAPI staining was performed under a confocal fluorescence microscope and multi-channel (α-actinin and titin with DAPI) immunofluoresence images were taken for each cell treatment. For each image of NRVM in different mitotic stages found, the extent of α-actinin and titin disassembly were scored [0, intact sarcomeric proteins; 1, low (20–40% of sarcomere area) disassembly; 2, medium (50–70% of sarcomere area); 3, high, disassembly (>80% of sarcomere area)]. The determination of mitotic stage was based on chromatin morphology.

### In silico identification of potential phosphorylation sites on α-actinin

Known phosphorylation sites for ACTN2 (the primary sarcomeric α-actinin) were identified using the PhosphoSitePlus database (www.phosphosite.org) [33]. Potential kinases for these sites were identified by NetPhosK 1.0 [34], NetworKIN 3.0 [35], and Musite [36], using rat ACTN2 as an input sequence (with 99% sequence homology to human and mouse). Default parameters were used for all predictors. Predikin 2.1 was used to identify residues in ACTN2 that are potentially phosphorylated by focal adhesion kinase 1 (FAK1), with the suggested default cutoff of 60 [37]. As suggested by the authors, prediction values made using the SDR method were used. For residues which SDR could not calculate a score, the average score yielded by the PANTHER and KSD methods was used.

### Cell cycle synchronization and fluorescence-activated cell sorting (FACS) of NRVM

Freshly isolated NRVM were cultured overnight before being subjected to a cell cycle synchronization protocol. NRVM were then exposed to 2 mM thymidine in culture medium for 24 hr. The medium was then removed and replaced with fresh medium. Eight hr later the medium was replaced with culture medium containing Ro3306 (9 μM, Tocris Bioscience) for 16 hr of exposure to pause cells at the G2/M checkpoint. 4 hr after release from Ro3306, cells were washed with PBS, trypsinized and labeled with Vybrant DyeCycle Violet (Invitrogen) for 5 min at room temperature, and subjected to the BD FACSAria III sorting after adding propidium iodide to exclude dead cells.

### Cell fractionation and Phos-tag PAGE assay

FACS-sorted synchronized NRVM (5X10^5^) in different cell cycle stages were pelleted by centrifugation and were processed to obtain total cellular protein, cytosolic or sarcomeric protein fractions. For total protein, cell pellets were resuspended in RIPA buffer (50 mM Tris-HCl [pH 8.0], 150 mM NaCl, 1% Nonidet P-40, 0.5% sodium deoxycholate, 0.1% sodium dodecyl sulfate) supplemented with 1% protease inhibitor cocktail (Sigma). For cytosolic fractions, pellets were resuspended in lysis buffer [25 mM Tris-HCI, pH 7.4, 5 μM EGTA, 2 mM EDTA,100 mM NaF and 1% protease inhibitor cocktail]. After freezing and thawing the samples 3 times in liquid nitrogen, samples were centrifuged at 10,000g for 5 min at 4°C and the supernatant was collected as the cellular cytosolic fraction. The sarcomeric fraction was obtained by resuspending the remaining pellet in RIPA buffer supplemented with protease inhibitor cocktail, centrifugation as above, and collection of the supernatant. The remaining RIPA-resistant pellet is typically considered as “cellular debris”. However, to account for the possibility that selective retention of α-actinin in these fractions could bias our results, for a number of samples the remaining pellet was resuspended and sonicated in 6x SDS-PAGE loading buffer (62.5 mM Tris-HCl pH 6.8, 1% SDS, 8% glycerol, 1.5% 2-mercaptoethanol, 0.005% bromophenol blue). These samples were electrophoresed by 10% SDS-PAGE under reducing conditions. BCA protein assay was performed to assure equal protein loading. For Phos-tag PAGE assays, 50 μM of Phos-tag acrylamide (Wako) and 10 μM MnCl_2_ were added in the preparation of 10% SDS-PAGE gels. Prior to the transfer of proteins, the Phos-tag gels were incubated with transfer buffer containing 5 mM EDTA for 20 min and subsequently washed with fresh transfer buffer for 15 min. Samples were electroblotted onto pre-wetted PVDF membranes (Bio-Rad Laboratories) and probed with the appropriate antibodies as for a conventional Western blot.

### Induction of mitotic exit in NRVM

Cultured NRVM were exposed to 2 mM thymidine for 24 hr, and then incubated with fresh media for 8 hr. Nocodazole (0.33 μM) was added for 16 hr to arrest cells at prometaphase. Then, the nocodazole-containing media was replaced with fresh media alone or with 9 μM Ro3306. MG132 (20 μM) or cycloheximide (10 μM) were also added in some experiments. Cells were fixed at 20, 40, 60 and 120 min for native α-actinin and titin immunofluorescence studies. For each time point and treatment condition 3000 cells were counted in low magnification (10X) images. A close-up image under 60X oil lens was also recorded for all identified mitotic myocytes.

## Results

### Disassembly of sarcomeric proteins occurs much faster than normal protein turnover

Sarcomere disassembly must occur for complete NRVM mitosis to occur. NRVM were fixed and stained for native α-actinin, and cells at different stages of spontaneously-occurring mitosis were identified based upon their chromatin morphology (Fig 1A). Because α-actinin is a Z-disk protein which is tightly incorporated into Z-disk structures, immunostaining of α-actinin in interphase cells shows an organized striated pattern (Fig 1Ai). The striated sarcomere structure, visualized by labeling for α-actinin, starts to disassemble at prophase and remains disassembled until late telophase. Interestingly, from late prophase extending to early telophase, immunostaining was dramatically enhanced but as a more diffuse pattern unassociated with striations (Fig 1Aii–vi). A reasonable explanation of this phenomenon is the increased availability of α-actinin immune epitopes in the cytosol upon disassembly of the densely-packed sarcomeric structure. This was observed to be such a robust and consistent phenomenon that we used the ratio of mean whole cell fluorescence intensity between a mitotic cell against an interphase cell, assessed in the same image field, as a quantitative index of sarcomere disassembly (Fig 1B). This ratio accurately reflected the degree of α-actinin disassembly (reduced visibility of defined Z-lines) for each individual mitotic cell assessed. Fully disassembled cells usually had a disassembly index close to 3. Non-mitotic cells consistently had values close to 1. The mean disassembly indices calculated from 20 randomly assessed cells from 3 unique cell isolation and cultures, for each different mitotic sub-phase, are shown in Fig 1B. There was a significant increase in the disassembly index from prometaphase through anaphase, consistent with the results in Fig 1A.

We also transduced cells with lentiviral vector bearing Halo-tagged α-actinin in order to monitor kinetic changes during the initiation of Z-disk disassembly during prophase using live cell imaging. Halo-tagged α-actinin was expressed and successfully incorporated into Z-disks, showing the same clear striation pattern as evident for native α-actinin (Fig 1C). The rate at which the striation pattern was lost when disassembly was initiated is remarkable. In early prophase cells, within 30 min or less there was a marked loss of the striated pattern of α-actinin while DNA condensed (Fig 1C and S1 Movie). The uniformly bright and clear Z-bands quickly blur and reduce in fluorescence intensity, and begin to dissolve, with the α-actinin at the periphery of the cell least affected. Unlike native α-actinin visualized by immunofluorescence staining (Fig 1A and 1B), disassembled, Halo-tagged α-actinin does not show the dramatic increase in its fluorescence signal, although a change from an orderly striated to a more diffuse pattern occurs.

**Fig 2:**
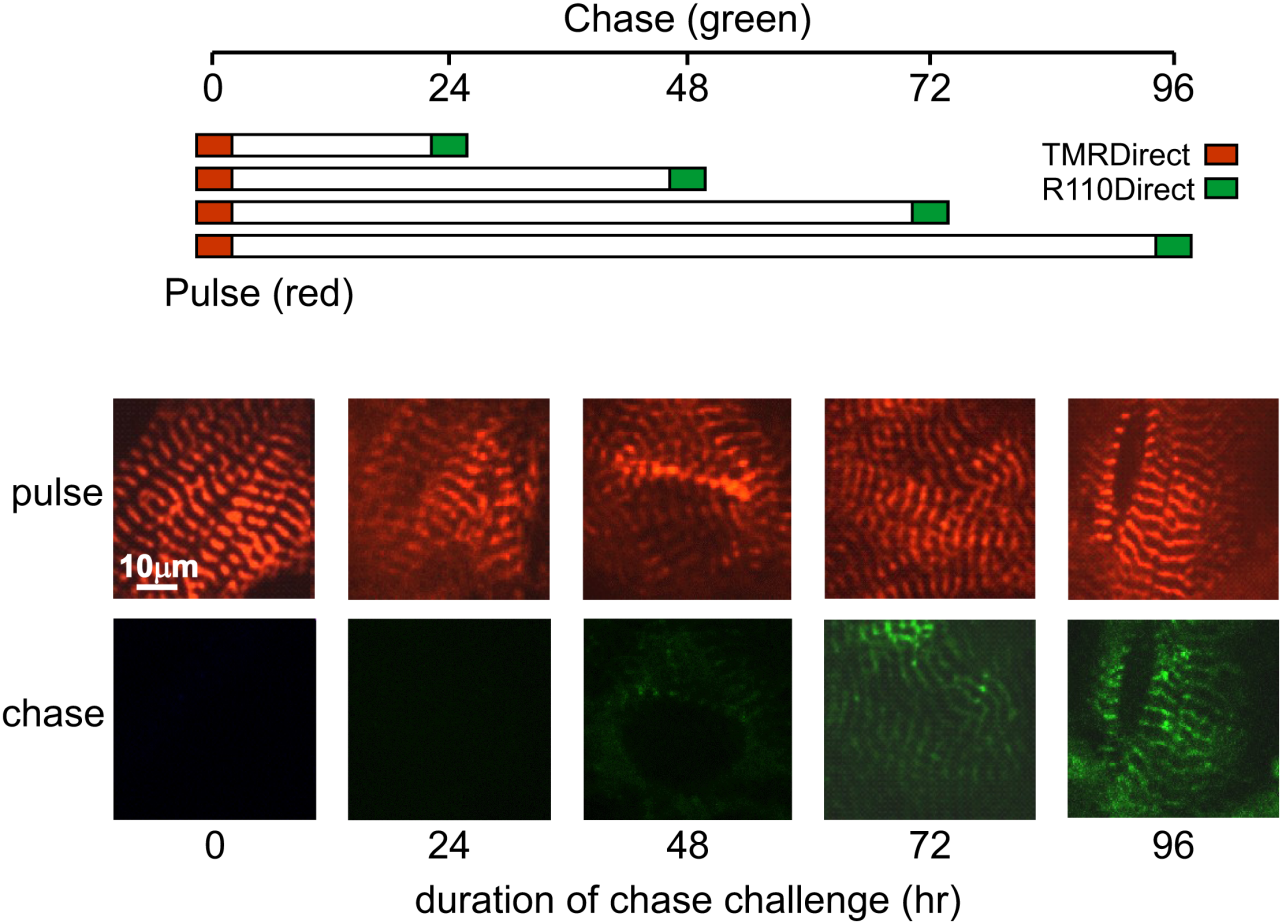
The turnover rate of α-actinin assessed in a pulse-chase experiment. NRVM were transduced with lentiviral vector harboring C-terminal HaloTag labeled α-actinin. 48 hr after transduction, cells were stained with excess TMRDirect (red fluorescence), a cell permeable ligand which forms a stable covalent bond with HaloTag α-actinin. At specified time points after TMRDirect staining as indicated, a different Halo-tag ligand (R110Direct, green fluorescence) was used to stain *de novo* synthesized HaloTag α-actinin. Both red and green fluorescence were assessed simultaneously. Any newly synthesized α-actinin in different time intervals after TMRDirect staining would be labeled with R110Direct. Representative images were taken under identical exposure parameters. Slight variations in background fluorescence between durations could be due to cell-to-cell variation, differing locations on cover slips, slight variation in depth of mounting media, or to changes in focus.

In order to rule out the possibility that the changes in α-actinin staining were due to normal protein turnover, a pulse-chase experiment was devised to estimate the physiological turnover rate of α-actinin. This was done by applying two different Halo-tag ligands (TMRDirect, red fluorescence; R110Direct, green fluorescence) at different times to the transduced cells with a schedule as indicated in Fig 2. Both ligands form a stable covalent bond with unbound C-terminal Halo-tagged α-actinin. TMRDirect ligand was applied at the start of the experiment so that it would occupy the α-actinin Halotag epitope. The R110Direct ligand was applied at 24 hr intervals after that. Consequently, any R110Direct ligand subsequently applied would not be bound unless new Halo-tagged α-actinin was synthesized. Thus, any Halo-tagged α-actinin newly synthesized after initial TMRDirect staining would be labeled by R110Direct. Cells were fixed at the end of the experiment and both red and green fluorescence were simultaneously assessed. Based on the changes of intensity of Halo-tagged α-actinin labeled with TMRDirect and R110Direct, the half-life of sarcomeric α-actinin for non-mitotic NRVM is estimated to be between 72–96 hr, a much longer time frame than that of α-actinin disassembly observed during mitosis (Fig 1C).

### Disassembly of sarcomeric proteins correlates with nuclear envelope breakdown

**Fig 3:**
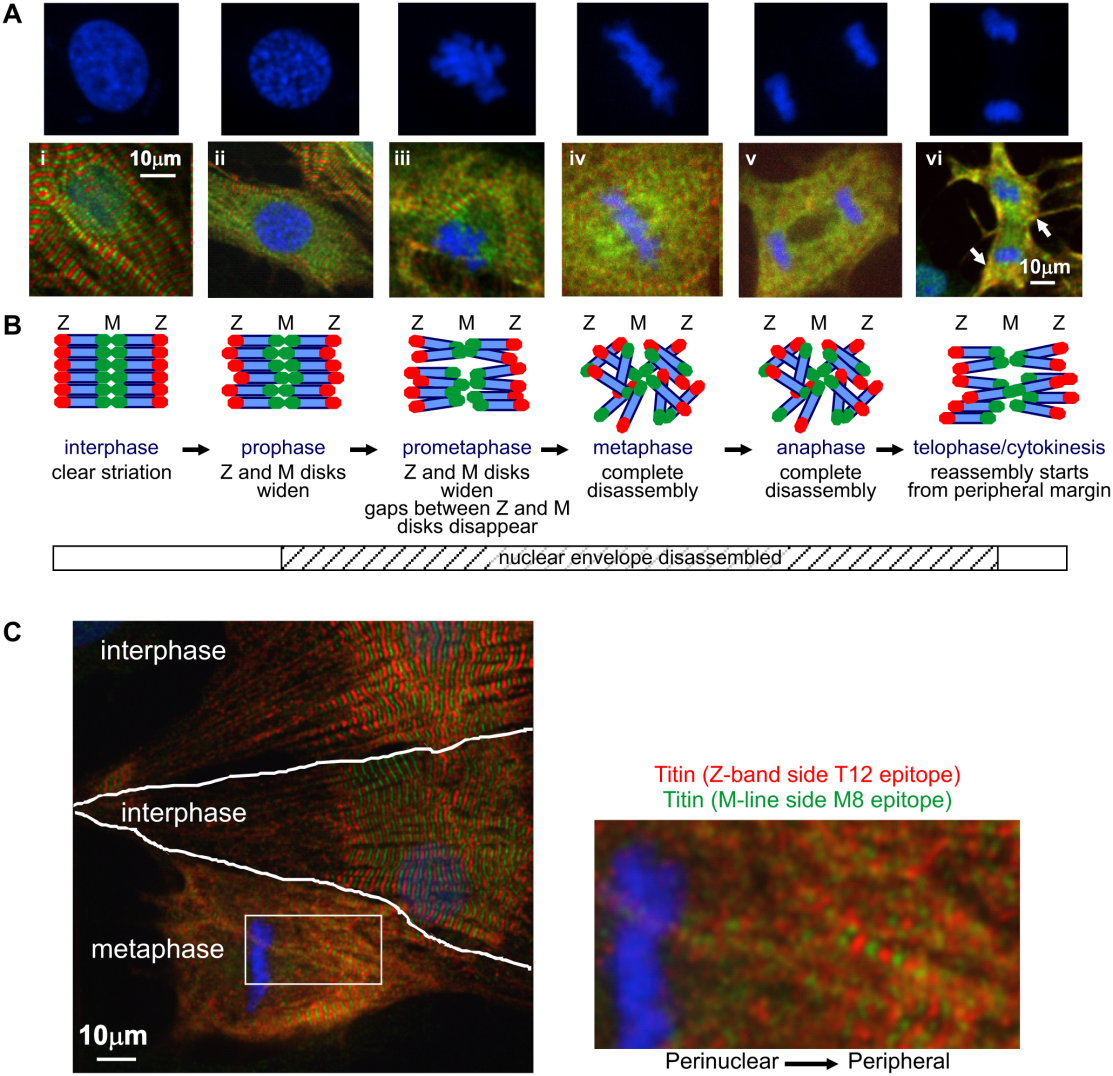
Pattern of titin disassembly during mitosis in NRVM. (A) Immunofluorescence staining of titin at both its N terminal (Z-disk, T12 antibody, red) and C terminal (M-line, M8 antibody, green) during mitosis. The clear alternating striation pattern of interphase cells (i) is disrupted starting from the end of prophase (ii) through prometaphase (iii). This is shown by a widening of both Z band and M line titin staining and the disappearance of gaps between the Z bands and M lines. Titin is almost completely disassembled by metaphase (iv) and remains disassembled through anaphase (v) until telophase (vi) when signs of reassembly appear in late cytokinesis. DNA is stained with DAPI (blue), and this is shown alone and in close-up above panels i to vi. (B) Proposed model of titin disassembly based on these observations. (C) Titin immunofluorescence image of a typical metaphase NRVM was shown along with two interphase cells. The drawn white lines show the border between cells. The boxed perinuclear region of titin undergoing disassembly is shown at higher magnification on the right. The transition titin disassembly pattern observed suggests that titin disassembly may start from the perinuclear zone and spread towards the peripheral margins of the cell.

Titin, the largest known mammalian protein, is an important multifunctional sarcomeric protein which serves as molecular spring and scaffold for the organized assembly of other myofilament proteins [25,26]. We stained native NRVM at both the Z-disk (T12) and M-line epitopes (M8) of the titin molecule [21]. Immunofluorescence of non-mitotic (interphase) cells shows an alternate red and green striation pattern typical of titin staining with these antibodies [21], with a small but clearly defined unstained dark space in-between (Fig 3Ai). We found in most observations that titin was disassembled at both its Z-disk and M-line ends before entering metaphase. Z-disk and M-line stained titin disassembled simultaneously in all cells we assessed. When titin disassembly initiates in prophase (Fig 3Aii) or prometaphase (Fig 3Aiii), the thickness of both Z-disk and M-line titin staining begins to widen along the longitudinal axis of the myofibril, while the unstained dark region that is normally apparent between the Z-disk and M-line quickly disappears (Fig 3Ai–iii, enlarged images shown in S1 Fig), ending up with the complete disruption of the orderly striated fluorescent signal (Fig 3Aiv–V). Fig 3B shows a spatial model of titin disassembly based on these observations. We propose that with α-actinin in the Z-disk being completely dissociated, the “unlocked” titin molecule starts to mobilize and move along the longitudinal axis of the sarcomere and the unstained region between the Z-disk and M-line quickly disappears. Thereafter, unanchored titin starts to completely disassociate from the sarcomere and is released into the cytosol, rendering a homogenous T12 and M8 epitopes fluorescence signal during anaphase (Fig 3Av).

Interestingly, disassembly of α-actinin begins promptly at the end of prophase, coincident with the start of nuclear envelope breakdown (Fig 1Aii). Ranging from the end of prophase to prometaphase, the disassembly of titin also starts, as shown by the aforementioned widening of the striated bands at both Z-disk and M-line sides (Fig 3Aii and 3Aiii). Early signs of α-actinin and titin reassembly begin in late telophase when new nuclear envelopes are formed for the two daughter nuclei (Figs 1Avi and 3Avi). Over multiple images, titin disassembly consistently started from the perinuclear zone and then spreads to the peripheral margin in metaphase (Fig 3Aiv and 3C). In contrast, titin reassembly starts from the peripheral margin in late telophase (Fig 3Avi).

### Titin disassembly occurs after α-actinin disassembly

**Fig 4:**
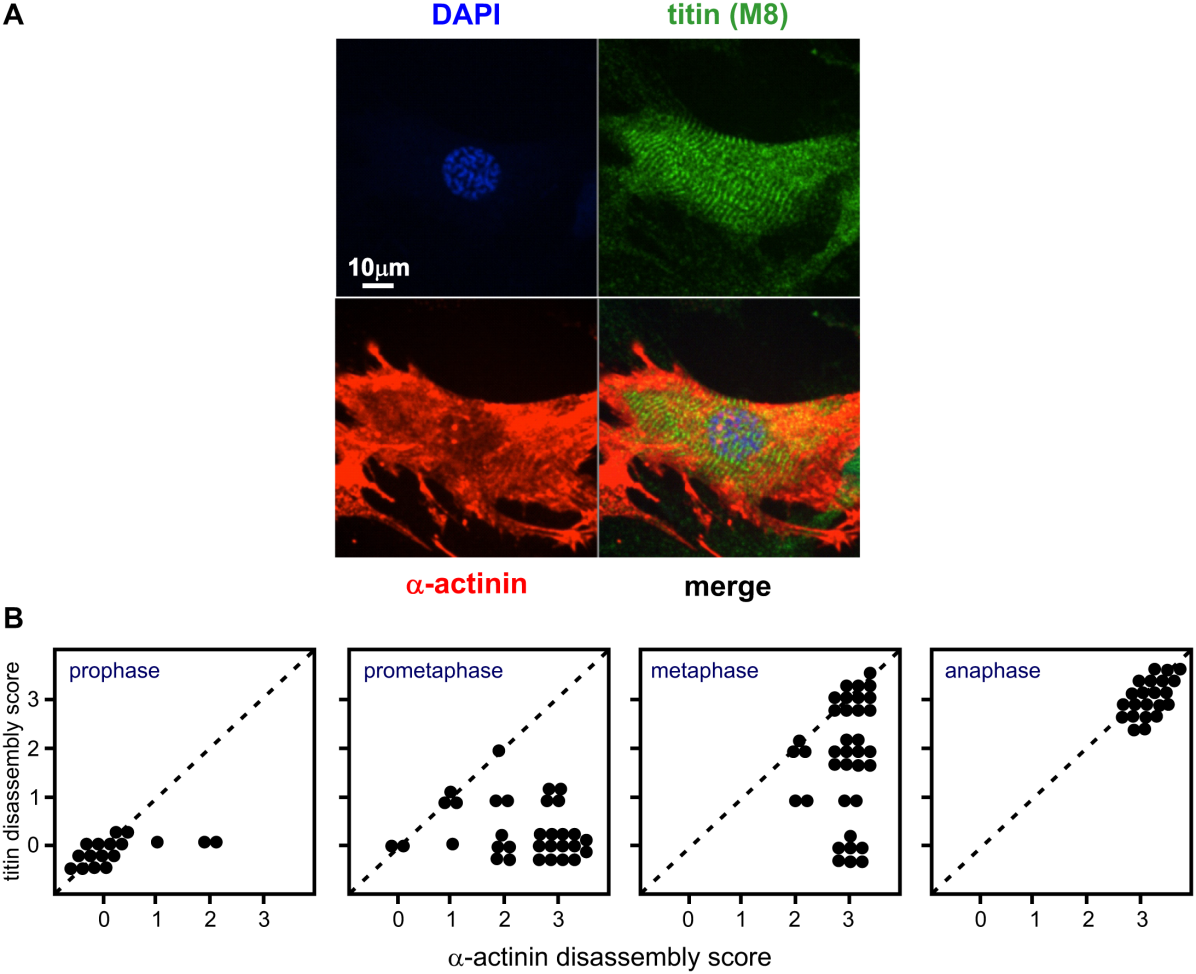
Disassembly of α-actinin precedes that of titin during mitosis. To delineate the possible sequential order of disassembly between α-actinin and titin during NRVM mitosis, triple staining of α-actinin (red), titin M8 epitope (green) and nuclear DNA (blue) was performed. (A) A typical prophase cell shows that α-actinin disassembly is well underway while titin remains intact. (B) For each image of mitotic NRVM in different mitotic stages found (screened from about 3000 NRVM per individual isolation over 5 isolations), α-actinin and titin disassembly scores were assessed. Mitotic stage determination was based on chromatin morphology. Disassembly score for both α-actinin and titin in each cell assessed is shown in two dimensional dot plots. Titin disassembly consistently occurs after α-actinin disassembly in timing.

To delineate the precise sequential order of disassembly of α-actinin and titin during myocyte mitosis, double staining of α-actinin (red) and titin M8 epitope (green) was performed. We consistently observed that titin disassembly generally began slightly after that of α-actinin in all cells imaged. A representative end prophase myocyte is shown in Fig 4A, in which α-actinin is mostly disassembled while titin still remains intact. To assess the timing difference between α-actinin and titin disassembly, the status of α-actinin and titin disassembly was scored (according to criteria described in the Methods section, “Simultaneous assessment of α-actinin and titin disassembly scores for protease inhibitor treatments”) for 107 randomly chosen mitotic cardiomyocytes. Generally both α-actinin and titin remained intact in prophase, although some α-actinin had already started to disassemble (Fig 4B). Through prometaphase and metaphase, both proteins started to disassemble, α-actinin to a significantly greater extent than titin (p < 0.0001 by chi-square test). By anaphase both proteins were fully disassembled.

### Sarcomere disassembly appears to be independent of major intracellular proteases

**Fig 5:**
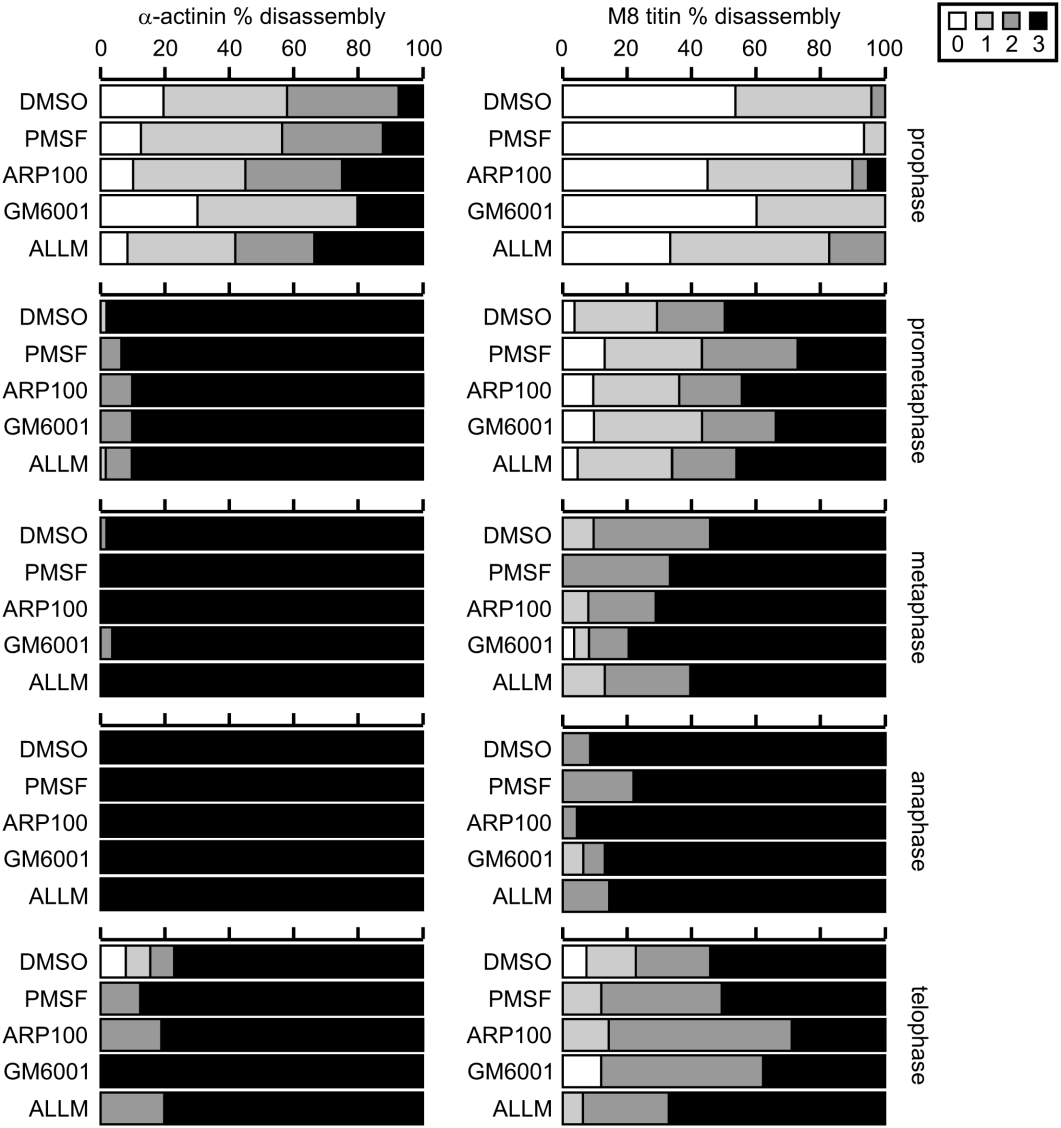
Protease inhibitors fail to interrupt sarcomere disassembly. NRVM were cultured in the presence of different protease inhibitors (PMSF, 20 μM; ARP100, 100 μM; GM6001, 5 μM or ALLM, 100 μM) for 4 hr before they were processed for immunofluorescence staining of α-actinin, titin (M8) and DAPI. α-actinin and titin disassembly were evaluated together for each single mitotic cell found in different treatment groups. The extent of α-actinin and titin disassembly were scored [0, intact sarcomeric proteins; 1, low (20–40% of sarcomere area) disassembly; 2, medium (50–70% of sarcomere area); 3, high, disassembly (>80% of sarcomere area)]. Imaging screening area and NRVM culture density were comparable between different treatment groups and a total of 584 mitotic NRVM over 13 independent isolations were found and recorded by confocal fluorescence microscope. The proportional distribution of different disassembly stages was plotted based on different mitotic stages and inhibitor treatments. Fisher's exact test was used to analyze the ranking data. None of the treatments show significant difference to vehicle control (p > 0.05).

Considering the rapid nature of the disassembly process, we hypothesized that intracellular proteases could be involved in sarcomere disassembly. Intracellular MMP-2 is the most important and abundant protease localized to specific sarcomere structures including the Z-disk in cardiomyocytes [21]. When activated in pathological conditions such as ischemia and reperfusion injury, it proteolyses both α-actinin [19] and titin [21]. Calpain is also reported to degrade some sarcomeric proteins [38]. We compared the effects of different classes of protease inhibitors including those of serine proteases, MMPs or calpains, on the timing and degree of disassembly of both α-actinin (Z-disk) and titin (M-line). We used the serine protease inhibitor PMSF, ARP-100 as a selective MMP-2 inhibitor [39], GM6001 as a pan-MMP inhibitor, and the calpain inhibitor ALLM. As some calpain inhibitors were shown to cross-inhibit MMP-2 activity we tested ALLM which does not [40]. We found that none of the protease inhibitors could significantly block or delay α-actinin or titin disassembly (Fig 5, Fisher's exact test; ranking analysis), suggesting a non-proteolytic nature of sarcomere disassembly. The proteasome inhibitor MG132 also had no effect on disassembly, instead inducing a high frequency of apoptosis despite our use of a lower concentration than previously reported [7] (S2 Fig). However, we were able to clearly identify 16 non-apoptotic cells in prometaphase or metaphase, all showing unchanged sarcomeric α-actinin disassembly. Also in contrast to that previous report, we found that Z-line and M-line epitopes of titin were disassembled simultaneously, rather than sequentially.

We observed rare (≤ 0.5%) mitotic NRVM with a near-intact sarcomere in late prophase to metaphase. This phenomenon was observed in both untreated NRVM and NRVM exposed to various protease inhibitors, with similar frequency. We interpret this as the naturally occurring cessation of sarcomere disassembly which may reflect the physiological transition of neonatal myocytes to non-proliferative, binucleated, mature myocytes.

### α-Actinin levels are increased at mitosis, with decreased phosphorylation of non-sarcomeric α-actinin

**Fig 6:**
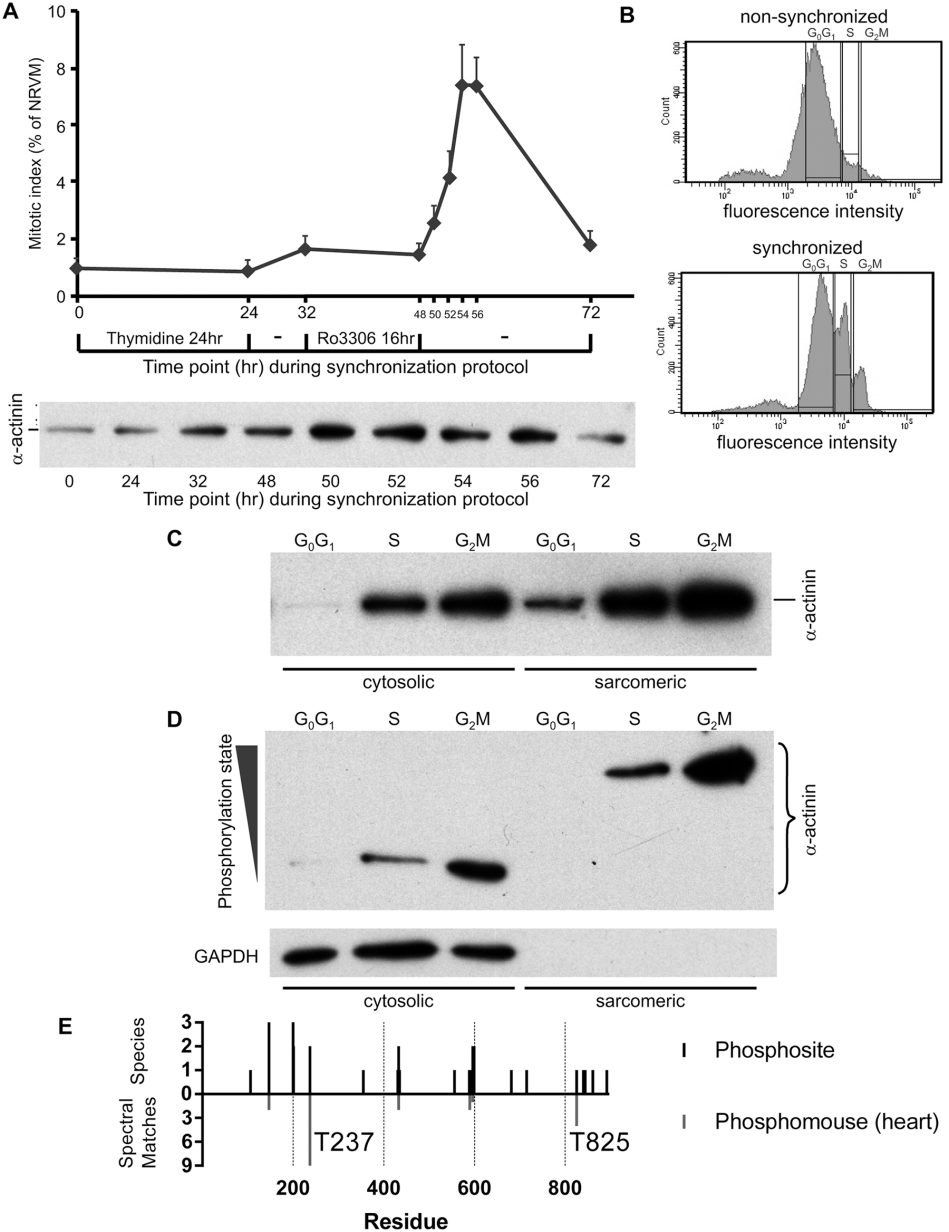
Analysis of cytosolic and sarcomeric α-actinin during mitosis. (A) Synchronized NRVM using thymidine and Ro3306 reveal increased α-actinin in RIPA buffer cell extracts in association with an increase in mitotic index. NRVM were cultured on 12 mm glass cover slips and synchronized with thymidine and 9 μM Ro3306 as indicated in the timeline. Immunofluorescence staining with α-actinin antibody and DAPI were performed to determine the mitotic index of NRVM at the different time points. A separate synchronization experiment was also performed in parallel on cells cultured in 6-well plates. At the same time points cells were lysed in RIPA buffer to obtain total cellular protein and this was analyzed for α-actinin by Western blot. The average mitotic index data from three independent experiments is shown. (B) Cell cycle analysis based on Vybrant DyeCycle Violet DNA staining was performed on non-synchronized NRVM (top panel) and synchronized NRVM (bottom panel). The high DNA content population (G_2_M population) increased in the cell cycle synchronized NRVM (4 hr after release from Ro3306) in comparison with non-synchronized NRVM. (C) Cytosolic and sarcomeric fractions of NRVM separated by FACS into different cell cycle stages based on DNA content were analyzed for α-actinin by Western blot analysis. (D) Phosphorylation status of cytosolic and sarcomeric α-actinin of FACS-sorted NRVM was determined using Phos-tag PAGE. GAPDH was used as a cytosolic marker. (E) Sarcomeric α-actinin phosphosites identified in large-scale phosphoproteomic screens. Upper bars show all sites reported in the *PhosphoSitePlus* database. The height of the bars corresponds to the number of species in which a given site was identified. Lower bars show phosphosites from mouse heart, with the height of the bar corresponding to the number of spectral matches, a semi-quantitative measure of phosphosite abundance. Residues of interest from the text are indicated.

To further explore the nature of changes in sarcomeric proteins during mitotic stages, we developed a cell cycle synchronization protocol (see Methods) to enrich mitotic NRVM. NRVM were harvested at different time points during the synchronization protocol for determination of their mitotic index and western blot analysis of α-actinin (Fig 6A). As predicted, the mitotic index increased following Ro3306 release. Of note, the level of α-actinin in total cellular protein extracts, normalized to protein content, was enhanced in parallel with the increased mitotic index of the cells (Fig 6A). In separate experiments performed in parallel, cells were labeled with Vybrant DyeCycle Violet after synchronization, and sorted by FACS. As illustrated in Fig 6B, NRVM in G_2_M phase were successfully enriched to about 8% of their total numbers in comparison to <2% in non-synchronized NRVM. Cells were collected at three different cell cycle stages (G_0_G_1_, S and G_2_M) and were then lysed for subsequent biochemical assays. The quantity of α-actinin in both the cytosolic and sarcomeric compartments was first assessed by conventional western blot. A dramatic increase in α-actinin levels (normalized to total cellular protein) in both compartments of cells in the G_2_M phase was observed (Fig 6C). Our results suggest that NRVM mitosis generates freely accessible (cytosolic) α-actinin which is released from intact sarcomeres. We hypothesized that a change in the phosphorylation status of α-actinin may contribute to this difference and therefore assessed it using Phos-tag PAGE. This analysis showed that α-actinin in the cytosolic fraction is less phosphorylated in comparison to that in the sarcomeric fraction (Fig 6D). Whether α-actinin was assessed on regular SDS-PAGE or Phos-tag gels, G_0_G_1_ cells consistently exhibited less α-actinin than S or G_2_M phase cells. We also assayed for α-actinin remaining in the RIPA-resistant pellet, expected to mostly consist of cellular debris, which was resuspended and sonicated in SDS buffer. Residual α-actinin in this fraction was also present in the greatest amounts in G_2_M cells, and showed a phosphorylation status similar to sarcomeric α-actinin (S3 Fig).

### In silico analysis of α-actinin phosphorylation

We wondered which kinase could be responsible for phosphorylation of α-actinin. Although we could not identify any published papers describing phosphorylation of sarcomeric α-actinin (ACTN2), several large-scale phosphoproteomic screens have identified phosphorylated ACTN2 residues. We used the PhosphoSitePlus database (www.phosphosite.org) [33] to identify known phosphorylation sites found within rat, mouse, or human ACTN2 (Fig 6E, upper bars). The amino acid sequence of rat, mouse and human ACTN2 is highly conserved, and the identification of the same phosphorylation sites across multiple species would therefore increase its certainty. One source of this phosphorylation data was a cross-tissue phosphoproteomics screen conducted in mice (https://gygi.med.harvard.edu/phosphomouse) [41]. This identified six phosphorylated peptides in the heart (S147, T237, S433, S590, S596 and T825; Fig 6E, lower bars).

There are a number of *in silico* methods for the prediction of phosphorylation sites and the responsible kinases [42]. We used NetPhosK [34], NetworKIN [35], and Musite [36] to predict potential kinases for the identified phosphosites. These 3 prediction algorithms were chosen to represent a cross-section of the different methodologies used to train the programs to predict phosphorylation sites (artificial neural networks, position-specific scoring matrices and support vector machines), different training data sets, as well as utilization of sequence information alone or in addition to structural information. Thus, agreement by these different methodologies would provide strong support for the potential of the given kinase to phosphorylated that specific residue. The different algorithms tended to vary in their predictions for each residue (Table 1, showing predictions for residues found to be phosphorylated *in vivo* as per Fig 6E). However, all three predictors suggested members of the casein kinase family, with high agreement for the C-terminal residue T825. Residue T237 (the phosphosite with the greatest spectral count in mouse hearts—Fig 6E) was predicted to be phosphorylated by kinases of the p38 MAPK or MAPK family by NetPhosK and Musite, respectively. Both NetPhosK and Musite identified potential CDK1 phosphorylation sites, but did not agree on the residue itself.

**Table 1:** In silico analysis to identify potential kinases of known phosphorylated residues in sarcomeric α-actinin (ACTN2).

		Kinase Predictors			Phosphosite Predictor
Position	Identity	NetPhosK	NetworKIN	Musite	Predikin-FAK*
106	Y	--	--	--	65.6
147	S	CKII	CaMKIIβ/δ/γ	--	69.8
200	Y	--	--	--	73.5
201	S	--	--	--	--
237	T	p38MAPK	--	CDK1/MAPK	--
355	S	--	PKCε/α/β/ζ / DMPK1	--	61.2
431	S	CDK1	--	--	68.6
433	S	CKII/CDK1	CK1δ	--	63.7
435	T	--	PKCε	--	--
556	T	PKG	CK1δ	--	--
589	Y	--	--	--	61.6
590	S	PKC	PKCα/ε/β/ζ	--	--
594	S	PKA	DMPK1	--	--
595	S	PKG	CaMKIIβ/δ	--	74.6
596	S	PKC	--	--	--
599	Y	INSR	--	--	82.5
681	Y	--	--	--	66.4
715	Y	--	--	--	70.8
825	T	CKII	CK1δ	CK2	70.1
840	S	PKC	PKCε/β/α/ζ	--	--
844	Y	--	--	--	67.7
861	Y	--	--	--	67.0
892	S	CKII	--	CK1	--

Slashes denote multiple predicted kinases for a given site (in order of likelihood)

--: no kinase passed the acceptance threshold for this site

*: Predikin score corresponds to likelihood of phosphorylation of the given site by FAK. Cut-off is 60.

CK: casein kinase. MAPK: mitogen-activated protein kinase. PK: protein kinase. INSR: insulin receptor. CaMKII: calcium/calmodulin-dependent protein kinase II

It has previously been shown that non-muscle/cytoskeletal α-actinin (ACTN1) is phosphorylated at Y12 by the focal adhesion kinase (FAK) [43]. Although Y12 was not identified as a phosphosite of sarcomeric α-actinin (ACTN2) *in vivo* (Fig 6E), it is nonetheless possible that this or other residues could be phosphorylated under certain circumstances. None of the kinase prediction programs that we used included FAK, so we investigated this using Predikin 2.1, which can make predictions for protein-specific phosphorylation sites of user-provided kinase sequences [37]. It predicted with a high likelihood that FAK1 could phosphorylate several of the known phosphorylated residues of sarcomeric α-actinin (Table 1), in addition to Y12 (SDR score of 70.3).

### Artificially-induced exit from mitosis in NRVM is accompanied by rapid reassembly of α-actinin in the sarcomere Z band independent of protein synthesis

**Fig 7:**
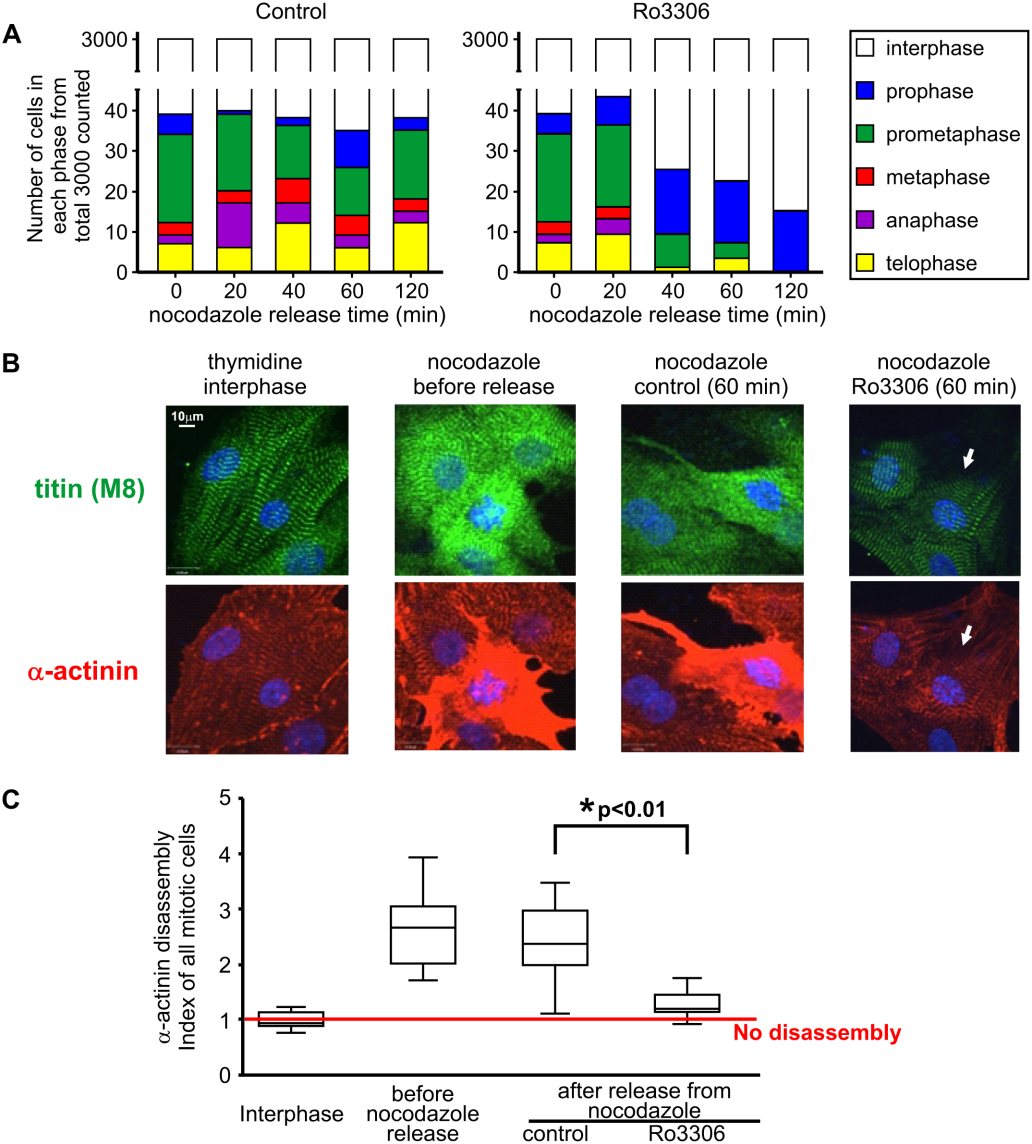
Induced exit from mitosis in NRVM is accompanied by rapid reassembly of α-actinin and titin. (A) After 16 hr of nocodazole exposure to enrich cells at prometaphase, fresh media alone (control) or with 9 μM Ro3306 replaced nocodazole-containing media. For each time point and treatment condition, 3000 NRVM were counted in the low magnification (10X) images and absolute numbers of cells in each mitotic sub-phase were plotted. (B) Immunofluorescence of a single representative cell chosen from each of: interphase cells after thymidine treatment, nocodazole enriched prometaphase cells, anaphase cells 1 hr after nocodazole release and prophase-like cells (indicated by arrows) after 60 min Ro3306 treatment following nocodazole release, are shown for both titin (M8) and α-actinin. (C) The α-actinin disassembly index was assessed for the different cell groups as shown above in (A) and (B). Analyzed cells were randomly chosen from three independent experiments. Data are presented as the median in a box and whisker plot. One way ANOVA followed by Tukey's multiple comparison test was performed. * p < 0.01 between control and Ro3306 groups is highlighted.

Given that proteases may not be responsible for changes in sarcomere structure during mitosis, we hypothesized that sarcomere disassembly could be a reversible process not dependent on protein synthesis. To test this concept, we forced NRVM to quickly exit mitosis using a strategy similar to one previously employed in immortalized cell lines, where cells arrested at prometaphase were treated with a selective CDK1 inhibitor, which resulted in chromosome decondensation and nuclear reformation [44]. We therefore exposed cultured NRVM to nocodazole (100 ng/ml) to arrest mitotic cells at prometaphase. Prometaphase cells were successfully enriched to 56% of total mitotic cells by nocodazole treatment (Fig 7A). After removing nocodazole by medium replacement, cells were cultured with or without the cyclin B1-CDK1 inhibitor Ro3306. Following release from nocodazole, NRVM cultures enriched with prometaphase cells pass through mitosis as indicated by the increased ratio of anaphase cells at 20 min and increased ratio of telophase cells 40 min after nocodazole release (Fig 7A left). However, when Ro3306 was added after nocodazole release (Fig 7A right), preventing cells from entering into prophase, prometaphase or metaphase, there was a reduction in the total number of mitotic cells within 40 min; among mitotic cells, there were fewer prometaphase cells and a dramatic increase in the proportion of prophase-like cells. By 60 min, a majority of the mitotic cells observed were prophase-like (Fig 7A right) containing both titin and α-actinin with an almost intact striated pattern (Fig 7B). By 120 min, only prophase-like cells could be observed. This is consistent with an ongoing process of nuclear reformation. Ro3306-induced exit from mitosis was consistently accompanied by the rapid reassembly of titin and α-actinin, within 1 hr (right side panels of Fig 7B). To study if the normal protein degradation pathway or *de novo* protein synthesis affect the reassembly processes, we performed two other experiments in parallel by adding either MG132 or cycloheximide 1 hr before inducing reverse mitosis with Ro3306. Neither blocking the common protein degradation pathway nor *de novo* protein synthesis showed any effects on sarcomere disassembly or reassembly (S4 Fig). Thus, sarcomere disassembly and reassembly in NRVM are not modulated by proteosomal protein degradation and are independent of new protein synthesis.

## Discussion

In this study we investigated the nature of sarcomere disassembly in NRVM and made several findings including: a) sarcomere disassembly appears to be independent of intracellular proteolysis, b) α-actinin disassembly precedes that of titin, suggesting that titin may disassemble secondary to the collapse of the Z-disk; c) α-actinin disassembly and reassembly were concurrent with the dissolution and reforming of the nuclear envelope, respectively; d) the level of dephosphorylated cytosolic, and phosphorylated sarcomeric, α-actinin increased during mitosis, e) inhibition of CDK1 induced the quick reassembly of the sarcomere, suggesting a role of CDK1 in the regulation of both disassembly and reassembly of the sarcomere and f) reassembly occurs without new protein synthesis.

We found no evidence for protein degradation during mitosis-induced sarcomere disassembly. All protease inhibitors tested, including the proteasome inhibitor MG132, failed to interrupt sarcomere disassembly. A previous study found that MG132 (20 μM) interfered with sarcomere disassembly in embryonic cardiomyocytes [7], whereas we found that the predominant effect of MG132 in our cultures was the induction of apoptosis. Although this complicated the interpretation of the results, we saw no evidence for delayed or interrupted sarcomere disassembly in non-apoptotic cells. A possible explanation for this discrepancy is the difference between embryonic and neonatal cardiomyocytes, the latter of which exhibit greatly decreased proliferative capacity in vitro. In fact, the authors suggested that it was the energetic costliness of the proteolytic disassembly-reassembly process that impedes cardiomyocyte division after birth. It is possible that, post-birth, the primary method of sarcomere disassembly may transition from a proteolytic process to one based on a reversible disassembly of the sarcomere. Another difference we observed was the simultaneous disassembly of both Z- and M-band epitopes of titin, which, in embryonic cardiomyocytes, were instead found to disassemble sequentially.

Another aspect of our study also supports a non-proteolytic nature of sarcomere disassembly. If sarcomere reassembly is based on the re-synthesis of intact sarcomeric proteins to replace proteolysed proteins, it is unlikely to be feasible because of the very fast nature of sarcomere reassembly that we observed in the artificially-induced rapid exit from mitosis induced by Ro3306 (Fig 7). Failure of the protein synthesis inhibitor cycloheximide and the proteasome inhibitor MG132 to interrupt Ro3306-induced reassembly of the sarcomere further strengthen this notion (S4 Fig). Also the fact that we found increased cytosolic free α-actinin associated with a greater mitotic index further suggests that protein degradation is not involved in sarcomere disassembly. Furthermore, we did not see any α-actinin cleavage products or degradation bands in fractions from cells in any cell cycle stage (S3A Fig).

Interestingly, we noticed that disassembly of sarcomeric α-actinin and titin starts at the end of prophase, coincident with the start of nuclear envelope breakdown. Early signs of reassembly of α-actinin and titin begin in late telophase when new nuclear envelopes are formed for each of the two daughter nuclei. Furthermore, multiple observations in our study consistently suggest that titin disassembly starts from the perinuclear zone and then spreads to the peripheral margin in metaphase. Moreover, titin reassembly starts from the peripheral margin in late telophase. These observations suggest that there may be some correlation between disassembly of the sarcomere with that of the nuclear envelope. A “disassembly signal” may be released from the nucleus when its envelope dissolves during late prophase. Reassembly could be accompanied by neutralization of that signal and the nuclear envelope returning anew to an intact status. CDK1 could therefore be a candidate for this putative disassembly signal, since it is activated in the nucleus by complexing with cyclin B1 in prophase [45,46], and should therefore be released into the cytosol upon disassembly of the nuclear envelope. Activated cyclin B1-CDK1 complexes function as a serine/threonine kinase, and phosphorylate hundreds of known target proteins including histone H1, nuclear lamins, centrosomal proteins, microtubule associated proteins [47] and potentially α-actinin (Table 1) to promote mitosis.

In silico analysis suggested that p38 MAPK could phosphorylate α-actinin. This is interesting in light of the fact that p38 MAPK inhibition facilitates the proliferation of adult cardiomyocytes [24]. p38 MAPK may function, in part, by keeping α-actinin phosphorylated, and hence in the sarcomeric form, whereas inhibition may facilitate its dephosphorylation and disassembly. However, sarcomere disassembly appears to progress normally in binucleating cells expressing wild-type levels of p38 MAPK [13]. Unfortunately, the current work did not differentiate between binucleation and proliferation, a distinction which is also lacking in much of the literature [48].

A yet to-be-identified phosphatase activated downstream of the cyclin B1-CDK1 cascade may be responsible for the dephosphorylation of sarcomeric α-actinin. One potential phosphatase is protein-tyrosine phosphatase 1B (PTP 1B), which is a CDK1 substrate [49] and has been shown to dephosphorylate non-sarcomeric α-actinin at Y12, leading to the dispersal of focal complexes at cell adhesion sites [43]. Y12 is also conserved in the sarcomeric α-actinin isoforms, indicating that PTP 1B may also dephosphorylate sarcomeric α-actinin. PTP 1B can also dephosphorylate the potential α-actinin kinase FAK1 (Table 1) to inactivate it [49].

Thus, we can hypothesize a scenario where phosphorylation by FAK and/or members of the p38 MAPK family maintain sarcomeric α-actinin in its phosphorylated state during interphase. With the onset of mitosis, CDK1 may activate PTP 1B which directly dephosphorylates α-actinin as well as inactivating FAK, impairing re-phosphorylation. The balance of α-actinin is therefore shifted towards the dephosphorylated state which, through unknown mechanisms, facilitates its disassembly. Given our observations that titin disassembly occurs slightly after that of α-actinin (Fig 4), it is possible that dissociation of Z-disk α-actinin initiates sarcomere disassembly.

The role of phosphorylation in the regulation of mitosis and cytokinesis is now gaining much attention [50]. Chen et al. [51] systematically investigated the possible mitotic roles of 117 protein phosphatases in Drosophila through RNAi and identified at least eight protein phosphatases including JNK MAP-kinase inhibitory phosphatase, PP2C and dual-specificity phosphatase Cdc25 to play important roles in the progression of mitosis. Other sarcomeric proteins have also been shown to be regulated by phosphorylation [52,53]. Future studies will be needed to confirm that changes to α-actinin phosphorylation state truly play causative roles in sarcomere dis- and re-assembly. These should include, as a start, investigations into potential roles played by p38 MAPK, FAK and CDK1 itself in α-actinin phosphorylation, and PTP 1B in its dephosphorylation.

In conclusion, sarcomere disassembly in the mitotic cardiomyocyte is not mediated by intracellular proteases but likely by cyclin B1-CDK1 mediated downstream signaling. Sarcomere disassembly coincides with an increase in α-actinin, in both soluble, cytosolic forms as well as insoluble, sarcomeric forms. Our study demonstrates that sarcomere disassembly is reversible, and that cytosolic and sarcomeric α-actinin have distinct phosphorylation states. This study provides additional clues to the puzzle of sarcomere disassembly during cardiomyocyte mitosis. Understanding these mechanisms may help to devise a means to facilitate sarcomere disassembly of mature cardiomyocytes and re-initiate cardiomyocyte proliferation in order to repair damaged myocardium.

## Supporting Information

S1 Fig Enlarged images of panels Ai, Aii, and Aiii from Fig 3. (TIF)

S2 Fig Typical apoptotic NRVM seen in MG132 treated cells. The red is α-actinin staining and green is titin stained at M8 epitope. Both sarcomeric proteins have lost their normal organization. DNA is stained with DAPI, and has condensed into compact patches against the nuclear envelope (pyknosis, typical of apoptosis), which appears discontinuous.
(TIF)

S3 Fig Comparison of α-actinin in cell debris fraction with cytosolic and sarcomeric fractions. (A) Cytosolic, sarcomeric and cell debris fractions of NRVM separated by FACS into different cell cycle stages based on DNA content were run out on a 10% SDS-PAGE gel, and analyzed for α-actinin by Western blot analysis. (B) Cell cycle analysis based on Vybrant DyeCycle Violet DNA staining for the samples shown above. (C) Comparison of α-actinin levels in the different cell cycle stages between total protein and cell debris fractions. (D) Phos-tag Western blots showing phosphorylation status of α-actinin present in the cell debris fraction vs. cytosolic and sarcomeric fractions for two experiments. Note that the cytosolic and sarcomere fractions of the lower experiment were shown in Fig 6D.
(TIF)

S4 Fig Induced exit from mitosis and the sarcomere reassembly is not affected by inhibition of the proteasome degradation pathway or new protein synthesis. NRVM enriched for prometaphase cells were exposed to Ro3306 as described in Fig 7. Experiments were performed in parallel with either the proteasome inhibitor MG132 (20 μM) or 10 μM cycloheximide, a protein synthesis inhibitor, added 1 hr before Ro3306. Immunofluorescence staining of sarcomeric α-actinin (AlexaFluor 568 nm) and DAPI was imaged using a 60X oil lens and a representative image for each treatment was shown for each of: interphase cells after thymidine treatment, nocodazole enriched prometaphase cells, anaphase cells 1 hr after nocodazole release and prophase-like cells (indicated by arrows) after 60 min Ro3306 treatment following nocodazole release.
(TIF)

S1 Movie Time lapse live-cell images show the rapid progress of α-actinin disassembly during prophase. 48 hr after transduction with a lentiviral vector harboring C-terminal HaloTag fused α-actinin, transduced NRVM were stained with cell permeable TMRDirect halo ligand (red) and Vybrant DyeCycle Violet (blue). A cell in early prophase was recorded over 1 hr by time lapse images taken at a rate of 1 frame/min and this repeats once during the video.
(WMV)
