# Supplementary figures and images for: Dynamic Alterations to α-Actinin Accompanying Sarcomere Disassembly and Reassembly during Cardiomyocyte Mitosis
Source: PLoS One. 2015 Jun 15;10(6):e0129176. doi: 10.1371/journal.pone.0129176 (PMC4467976; doi:10.1371/journal.pone.0129176)

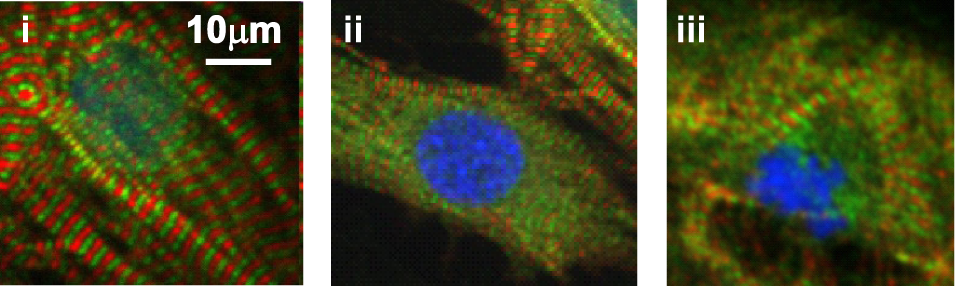

Supplement: S1 Fig — (TIF) [file pone.0129176.s001.tif]

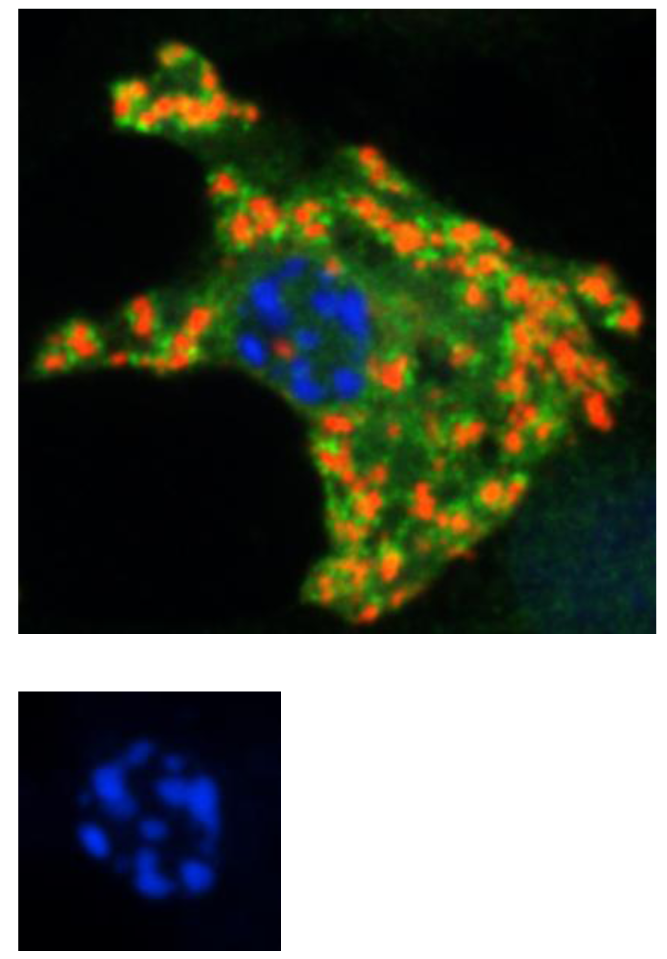

Supplement: S2 Fig — The red is α-actinin staining and green is titin stained at M8 epitope. Both sarcomeric proteins have lost their normal organization. DNA is stained with DAPI, and has condensed into compact patches against the nuclear envelope (pyknosis, typical of apoptosis), which appears discontinuous. (TIF) [file pone.0129176.s002.tif]

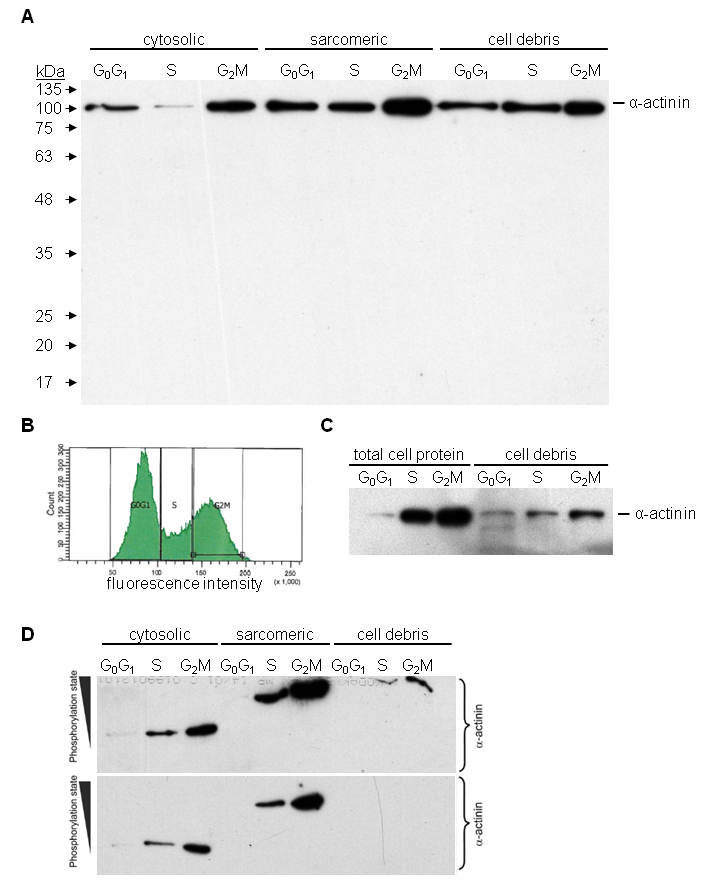

Supplement: S3 Fig — (A) Cytosolic, sarcomeric and cell debris fractions of NRVM separated by FACS into different cell cycle stages based on DNA content were run out on a 10% SDS-PAGE gel, and analyzed for α-actinin by Western blot analysis. (B) Cell cycle analysis based on Vybrant DyeCycle Violet DNA staining for the samples shown above. (C) Comparison of α-actinin levels in the different cell cycle stages between total protein and cell debris fractions. (D) Phos-tag Western blots showing phosphorylation status of α-actinin present in the cell debris fraction vs. cytosolic and sarcomeric fractions for two experiments. Note that the cytosolic and sarcomere fractions of the lower experiment were shown in Fig 6D. (TIF) [file pone.0129176.s003.tif]

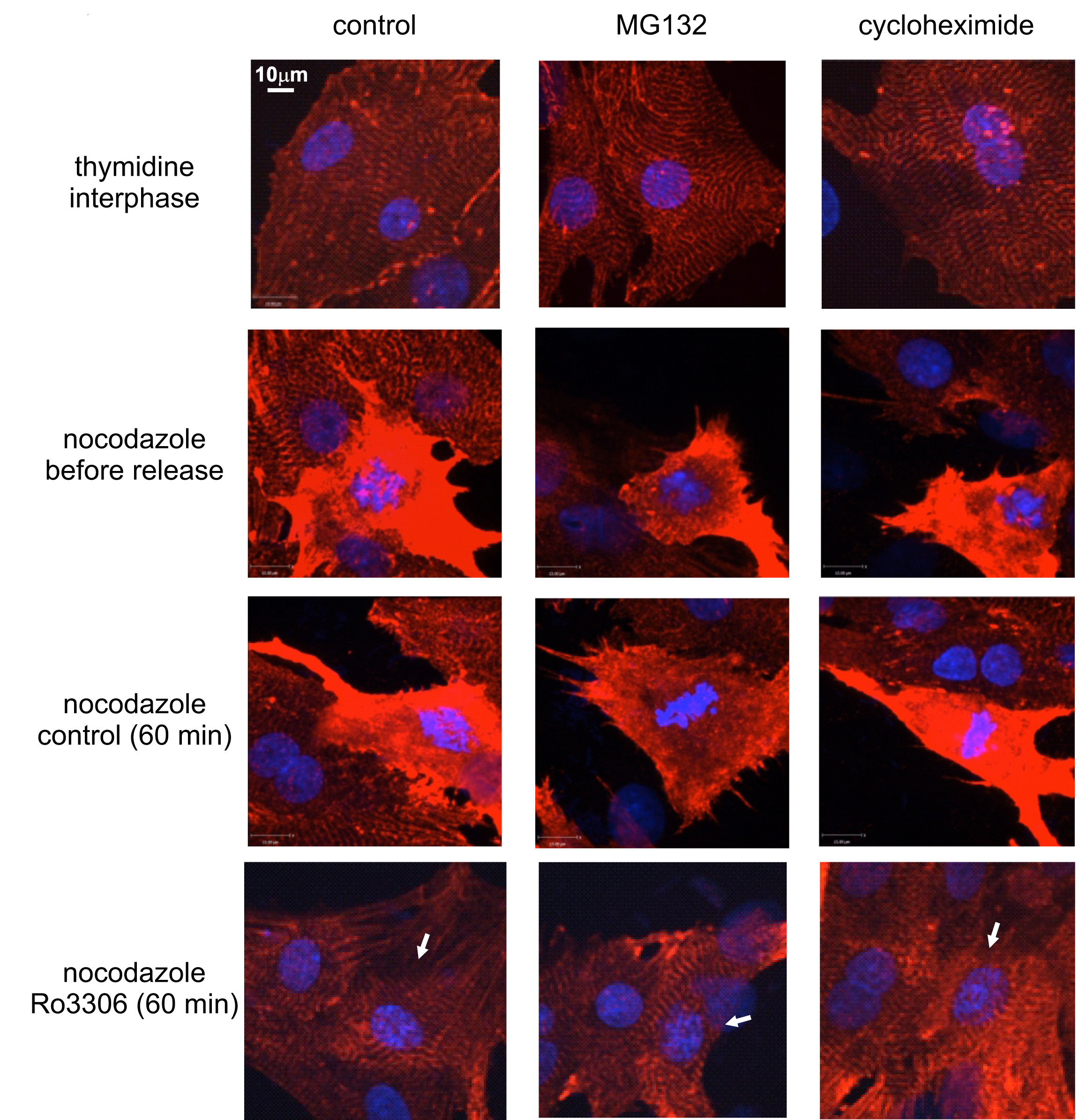

Supplement: S4 Fig — NRVM enriched for prometaphase cells were exposed to Ro3306 as described in Fig 7. Experiments were performed in parallel with either the proteasome inhibitor MG132 (20 μM) or 10 μM cycloheximide, a protein synthesis inhibitor, added 1 hr before Ro3306. Immunofluorescence staining of sarcomeric α-actinin (AlexaFluor 568 nm) and DAPI was imaged using a 60X oil lens and a representative image for each treatment was shown for each of: interphase cells after thymidine treatment, nocodazole enriched prometaphase cells, anaphase cells 1 hr after nocodazole release and prophase-like cells (indicated by arrows) after 60 min Ro3306 treatment following nocodazole release. (TIF) [file pone.0129176.s004.tif]
